# Supplementary material for: Ultrafast nematic-orbital excitation in FeSe
Source: Nat Commun. 2019 Apr 29;10:1946. doi: 10.1038/s41467-019-09869-5 (PMC6488589; doi:10.1038/s41467-019-09869-5)
Supplement: Supplementary file 1 — Supplementary Information [file 41467_2019_9869_MOESM1_ESM.pdf]

Supplementary Information for

Ultrafast nematic-orbital excitation in FeSe

T. Shimojima[1,2], Y. Suzuki[2], A. Nakamura[2], N. Mitsuishi[2], S. Kasahara[3], T. Shibauchi[4], Y. Matsuda[3], Y. Ishida[5], S. Shin[5] and K. Ishizaka[1,2]

[1]RIKEN Center for Emergent Matter Science (CEMS), Wako 351-0198, Japan

[2]Quantum-Phase Electronics Center (QPEC) and Department of Applied Physics, The University of Tokyo, Tokyo 113-8656, Japan

[3]Department of Physics, Kyoto University, Kyoto 606-8502, Japan

[4]Department of Advanced Materials Science, The University of Tokyo, Kashiwa, 277-8561, Japan

[5]Institute for Solid State Physics (ISSP), The University of Tokyo, Kashiwa, 277-8581, Japan.

Supplementary Note 1: Laser ARPES on detwinned FeSe in thermal equilibrium

Here we show the ARPES data[1] which was used to draw the schematic band structures in Fig. 1**b** and **d**. Supplementary Figure 1**a** and **b** show the experimental geometries used for the laser-ARPES measurements in thermal equilibrium. In the geometry in Supplementary Fig. 1**a** (1**b**), the strain direction is parallel (perpendicular) to the detector slit and the momentum along k_x (k_y) is measured. Here, *xz* (*yz* and *xy*) orbitals have even (odd) symmetry with respect to the mirror plane and can be detected by *p* (*s*)-polarized probe laser. Similarly, *yz* (*xz* and *xy*) are detected by *p* (*s*)-polarized laser in the geometry in Supplementary Fig. 1**b** which measures along k_y. The observable orbital characters are also indicated. Here, we can focus mainly on the *xz* and *yz* orbitals, since the photoelectron intensity of the *xy* orbital is quite weak near E_F in the ARPES measurements on FeSe[1,2].

In the tetragonal phase, FeSe exhibits a nearly circular Fermi surface around the Γ point as depicted in Fig. 1**a**. Along k_x (k_y), the band forming the Fermi surface is known to have the *yz* (*xz*) orbital component, which can be confirmed by the contrast of the polarization-dependent ARPES intensity indicated in the square boxes in Supplementary Fig. 1**c** and **d**. In the orthorhombic (nematic) phase, on the other hand, the momentum-dependent orbital polarization[1] modifies the shape of the Fermi surface into an elliptical one as shown in Fig. 1**c**, and the k_{Fx} and k_{Fy} become inequivalent as indicated by the black double-headed arrows in Supplementary Fig. 1**e** and **f**. At the same time, the light polarization dependence inside the square boxes now shows that the orbital components at the k_F's are mixed, especially along k_x, the shorter axis (Supplementary Fig. 1**e**). The band dispersions and the orbital characters were reproduced by the calculations including both the orbital non-equivalency and the spin-orbit coupling[1] as shown in Fig. 1**d**.

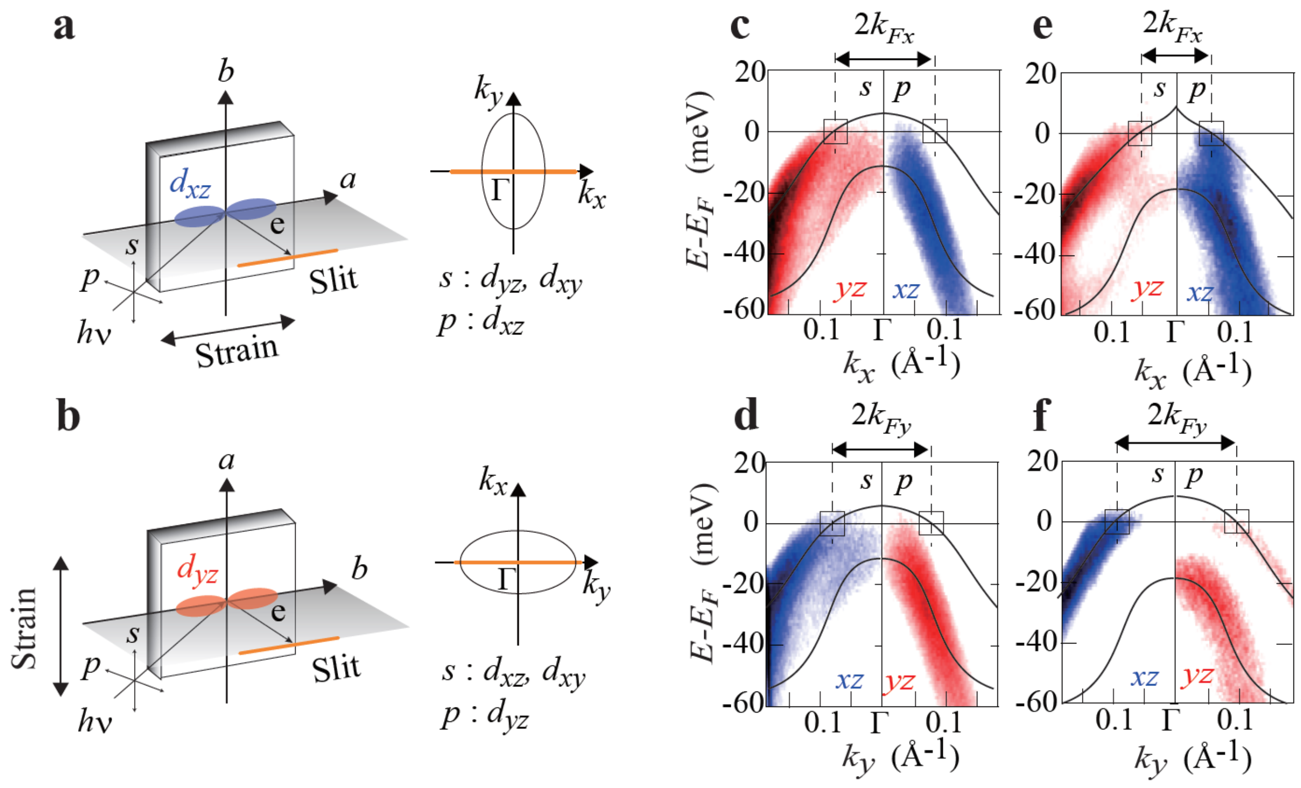

Supplementary Fig. 1. Experimental geometries for ARPES and *E*-*k* images of detwinned FeSe. a,b, The experimental geometries for polarization-dependent laser ARPES[1] in thermal equilibrium. In the geometry in **a** (**b**), orthorhombic *a* (*b*) axis is parallel to the detector slit. Gray plane represents a mirror plane of the orthorhombic lattice. We used the linear polarization *s* (*p*) which is perpendicular (parallel) to the detector slit. Momentum cut is shown in each geometry with the orange line. Observable orbital characters are also indicated for each polarization. **c**, Band dispersions along k_x axis around the Γ point of the detwinned FeSe obtained at 160 K with the *s*- and *p*-polarized laser ($h\nu$ = 5.9 eV). Black curves represent hole band dispersions. Dotted lines highlight the position of k_F. **d**, The same as **c** but along k_y. **e,f,** The same as **c** and **d** but obtained at 30 K.

Supplementary Note 2: Momentum dependence of the orbital characters in the hole Fermi surface around the Γ point

Here we discuss the momentum dependence of the orbital characters in the hole Fermi surface for understanding the redistribution of the orbital components across the nematic order. Supplementary Figures 2**a** and **b** are the Fermi surface-angle (θ) dependence of the orbital components in the tetragonal and orthorhombic phases, respectively, obtained from the calculations based on the *d*-*p* model[1] assuming the spin-orbit coupling and orbital non-equivalency, which exhibits the Fermi surface shape similar to that obtained by previous laser ARPES[1]. While the orbital components other than *xz* and *yz* orbitals were also mixed (~20 %) in the hole Fermi surface both below and above the nematic order, we focus on *xz* and *yz* orbitals for discussing the temperature dependence related to the nematic order.

In the tetragonal phase (Supplementary Fig. 2**a**), the band forming the Fermi surface has the dominant contribution of *yz* (*xz*) orbital along k_x (k_y). Here, *k*-dependent orbital characters keep the C_4 symmetry. In the orthorhombic phase (Supplementary Fig. 2**b**), the elliptical Fermi surface is composed of both *xz* and *yz* orbitals along k_x axis due to the orbital polarization, while that along k_y axis exhibits almost *xz* character. These θ dependences of the orbital components are qualitatively consistent with the polarization-dependent ARPES results shown in Supplementary Fig. 1**c** - **f**.

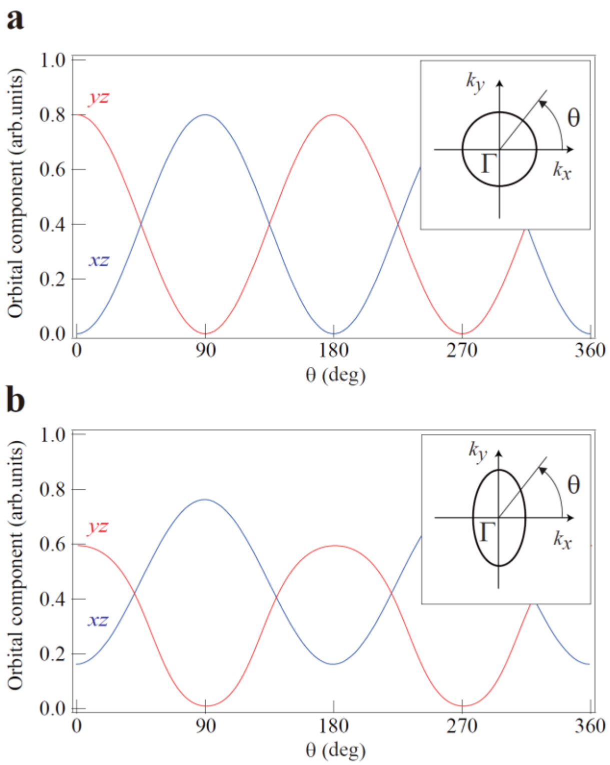

Supplementary Fig. 2. Calculated orbital characters of the Fermi surface around the Γ point of FeSe.

a,b, The Fermi surface-angle (θ) dependence of the orbital components in the tetragonal and orthorhombic phases, respectively. The blue (red) curves represent orbital component for the *xz* (*yz*) orbital. The inset shows the definition of the Fermi surface angle θ.

Supplementary Note 3: Experimental geometries for TARPES on detwinned FeSe

In Supplementary Figure 3**a**, we show the detwinning device made for TARPES measurements. Single crystals (2 mm × 4 mm × 0.05 mm) were cut along the orthorhombic axes and pulled along one of the axes by tightening the screw. The longer *a* axis is then aligned parallel to the direction of the tensile strain below T_s. Supplementary Figures 3**b** and **c** show the experimental geometries used for the TARPES measurements. In the geometry in Supplementary Fig. 3**b** (3**c**), the strain direction is set parallel (perpendicular) to the detector slit and the momentum along k_x (k_y) is measured. Here, we consider a mirror plane spanned by the surface normal and the detector slit. When using the *s*-polarized light, only the orbitals of even parity with respect to the mirror plane, i.e. *xz*, is observed due to the selection rule. Similarly, only *yz* is detected by the *s*-polarization in the geometry in Supplementary Fig. 3**c** which measures along k_y. Note that the selection rule for *p*-polarization holds only at the Γ point (*yz* for Supplementary Fig. 3**b** and *xz* for Supplementary Fig. 3**c**), by considering the other mirror plane spanned by the surface normal and the orthorhombic axis perpendicular to the detector slit. However, we can confirm that the orbital-selective observation remains capable also away from the Γ point, by directly comparing with the *E*-*k* images obtained by another laser-ARPES ($h\nu$ = 5.9 eV) system with a higher symmetry configuration (Supplementary Note 1). Owing to the orbital selectivity, we can separately discuss the *xz* and *yz* orbitals in the present TARPES measurements on FeSe.

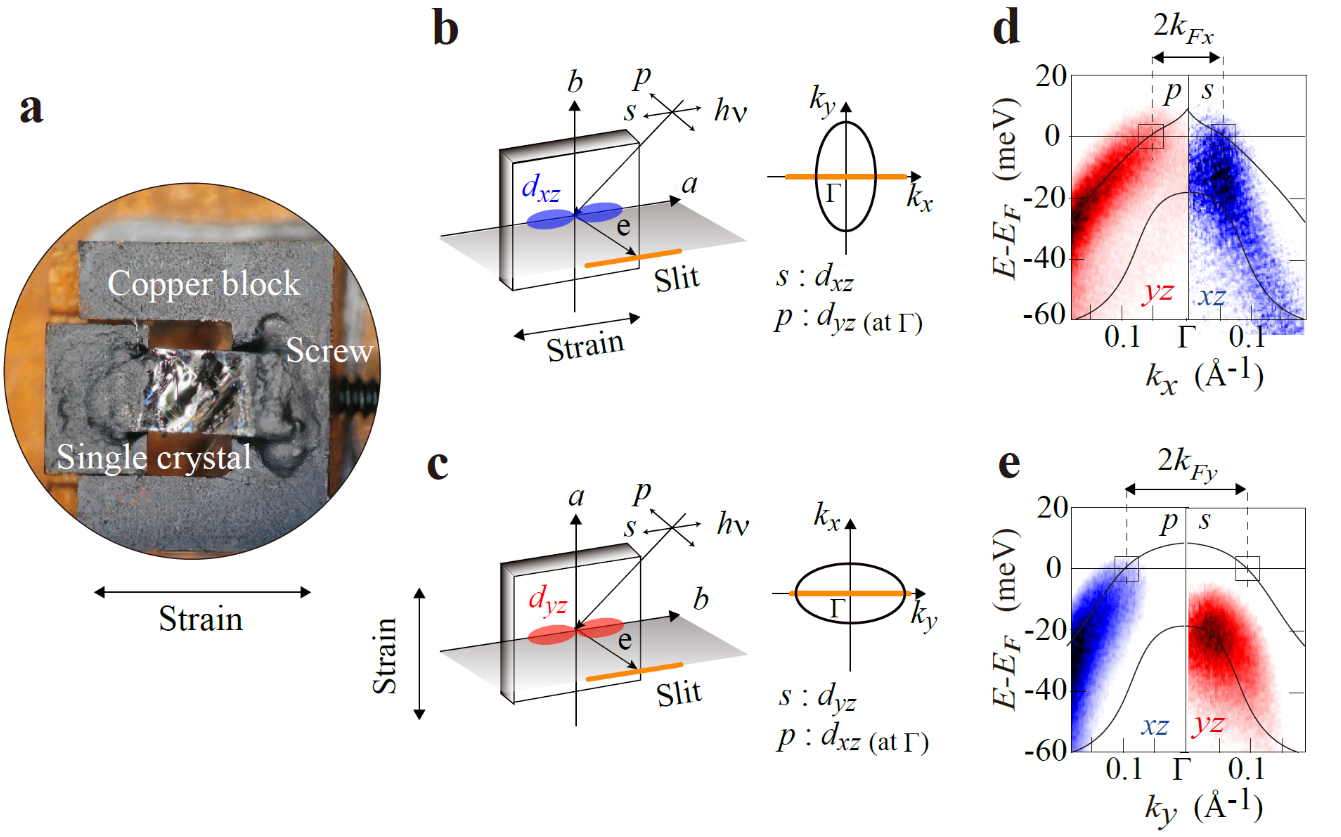

Supplementary Fig. 3. Experimental geometries for TARPES and *E*-*k* images of detwinned FeSe before photo-excitation. **a**, Detwinning device with the strained single crystal of FeSe. **b,c**, The experimental geometries for TARPES where the detector slit is parallel to the orthorhombic *a* and *b* axis, respectively. Gray plane represents a mirror plane of the orthorhombic lattice. The linear polarization *s* is parallel to the detector slit. Momentum cut is shown for each geometry with the orange line. Observable orbital characters are also indicated for each polarization. **d**, Band dispersions along k_x axis around the Γ point of the detwinned FeSe obtained at 20 K with the *s*- and *p*-polarized probe laser ($h\nu = 5.9$ eV) in the geometry of **b**. Black curves represent hole band dispersions. Dotted lines highlight the position of k_F. **e**, The same as **d** but along k_y in the geometry of **c**.

Supplementary Note 4: Carrier relaxation dynamics for *xz* electrons obtained by different linear polarizations of probe laser.

In Supplementary Figure 4**a**, **b,** we show the EDCs around the Γ point for *xz* electrons obtained by *p*-polarized probe laser along k_y, and *s*-polarized probe laser along k_x in the strong-excitation regime (F = 220 μJcm^{-2}) at 20 K, respectively. In both cases, we observe a non-monotonic relaxation of photo-excited *xz* electrons ($E_F < E < 10$ meV) which keep increasing from t = 120 fs to 700 fs. The $\Delta I(t)$ for *xz* obtained by *s*-polarization is almost identical to that of *p*-polarization as shown in Fig. 3g. We thus confirm that the retarded maximum in $\Delta I(t)$ solely depends on the orbital character, not on the experimental configurations such as probe-laser polarizations (*p* or *s*) and probed momentum directions (k_x or k_y).

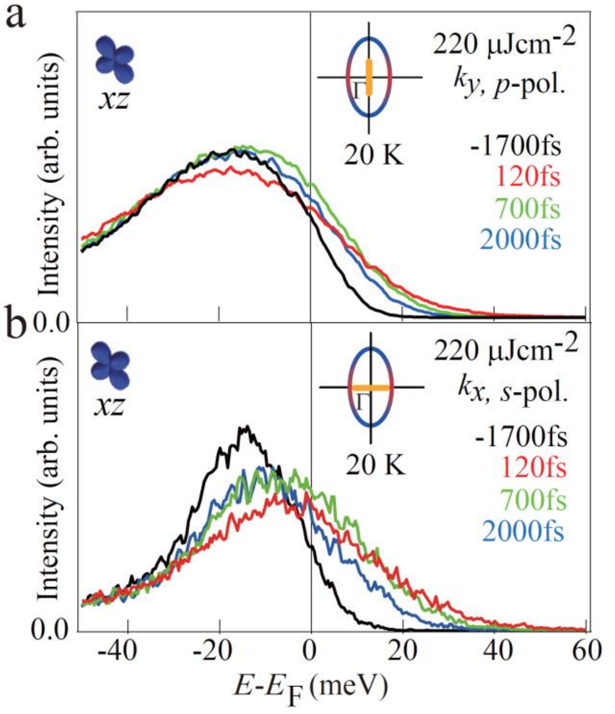

Supplementary Fig. 4. The EDCs around the Γ point probed by different probe laser polarizations in the strong-excitation regime. a, Time dependence of the EDCs around the Γ point ($k_y = 0.0 \pm 0.04$ Å^{-1}) for *xz* electrons obtained by *p*-polarized probe laser in the strong-excitation regime (F = 220 μJcm^{-2}) at 20 K. The orange bar in the inset shows the integrated momentum region for obtaining the EDCs. **b**, The same as **a** but obtained by *s*-polarized probe laser along k_x.

Supplementary Note 5: Temperature dependence of the carrier relaxation dynamics for *xz* and *yz* electrons in the strong excitation regime.

In Supplementary Figure 5**a, b,** we show the EDCs around the Γ point ($k_y = 0.0 \pm 0.04$ Å^{-1}) for *xz* electrons obtained by *p*-polarized probe laser in the strong-excitation regime (F = 220 μJcm^{-2}) at 20 K and 160 K, respectively. The photo-excited tail intensity of EDCs ($E_F < E < 10$ meV) at 160 K monotonically increases after photo-excitation. The indication of the retarded maximum in $\Delta I(t)$ is absent in the tetragonal phase.

In Supplementary Figure 5**c**, **d**, we also show the EDCs around the Γ point ($k_x = 0.0 \pm 0.04$ Å^{-1}) for *yz* electrons obtained by *p*-polarized probe laser in the strong-excitation regime (F = 220 μJcm^{-2}) at 20 K and 160 K, respectively. At 160 K, the line shape and time dependence of the EDCs for *yz* (Supplementary Fig. 5**d**) are almost comparable to those for *xz* (Supplementary Fig. 5**b**) reflecting the tetragonal symmetry. We thus confirm that the non-monotonic carrier relaxation for *xz* electrons appears only in the nematic phase.

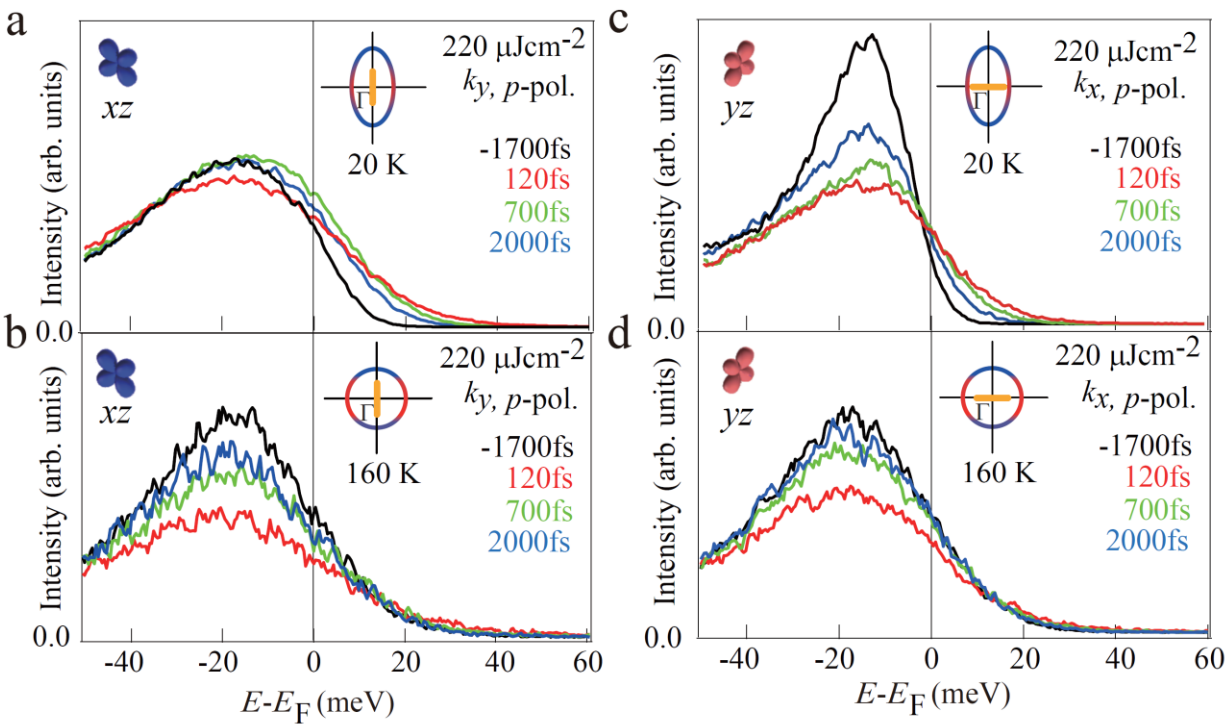

Supplementary Fig. 5. Temperature dependence of the EDCs around the Γ point in the strong-excitation regime. a, Time dependence of the EDCs around the Γ point (k_y = 0.0 ± 0.04 Å^{-1}) for *xz* electrons obtained by *p*-polarized probe laser in the strong-excitation regime (F = 220 μJcm^{-2}) at 20 K. **b,** The same as **a** but at 160 K. **c**,**d**, The same as **a** and **b** but for *yz* electrons obtained along k_x.

Supplementary Note 6: Fluence dependence of the time constants obtained from the fitting procedures on the $k_{Fy}(t)$ curves.

We show the time constants obtained from the fitting analysis on the $k_{Fy}(t)$ for several F values assuming the function $k_F(t) = k_{F1}\exp(-t/\tau_1) + k_{F2}\exp(-t/\tau_2) + k_{F3}\exp(-t/\tau_3)\cos(2\pi t/t_p)$ as summarized in the Supplementary Table 1. For F = 40, 80 and 170 μJcm^{-2}, we assumed the double exponential components.

F (μJcm^{-2})	τ_1 (ps)	τ_2 (ps)	τ_3 (ps)	t_p (ps)
430	0.40	≥ 80	0.40	0.6
330	0.45	≥ 80	0.55	0.8
240	0.68	≥ 80	0.60	1.2
220	0.83	≥ 80	0.55	1.4
170	0.83	≥ 80	-	-
80	0.8	≥ 80	-	-
40	0.85	≥ 80	-	-

Supplementary Table 1. Time constants extracted from the $k_{Fy}(t)$ curves.

Supplementary Note 7: Estimation of the transient electronic temperature.

Here we estimate the electronic temperature (T_e) from the fitting analysis of the momentum-integrated EDCs. In general, T_e should be estimated by using the momentum-integrated EDC spectrum which represents the total density of states multiplied by the Fermi-Dirac function further convoluted by the instrumental resolution function. We integrated the EDCs of ARPES on *xz* from 0.0 Å^{-1} to 0.17 Å^{-1} along k_y, and fitted by a FD function convoluted by the gaussian of energy resolution (20 meV), assuming the constant density of states neat E_F (Supplementary Fig. 6**a**). After the photoexcitation of 220 μJcm^{-2}, T_e reaches 88 ±2 K at 120 fs. Then, it shows a rapid decrease in < 1 ps and remains nearly constant at 45 K for t > 3000 fs (Supplementary Fig. 6**b**), which is considerably lower than T_s = 90 K.

According to the two-temperature model[3], elevated T_e approaches a constant value after the rapid relaxation *via* the electron-lattice coupling. There, the quasi-equilibrium state is realized, where the temperatures of electrons and lattice become equivalent. This behavior has been indeed discussed in the ultrafast optical measurements of the iron-based superconductors[4]. The maximum lattice temperature is thus expected to be ~45 K in the present TARPES case. These analyses suggest that the electronic nematic order gets dissolved in the ultrafast regime while the lattice well maintains the orthorhombicity.

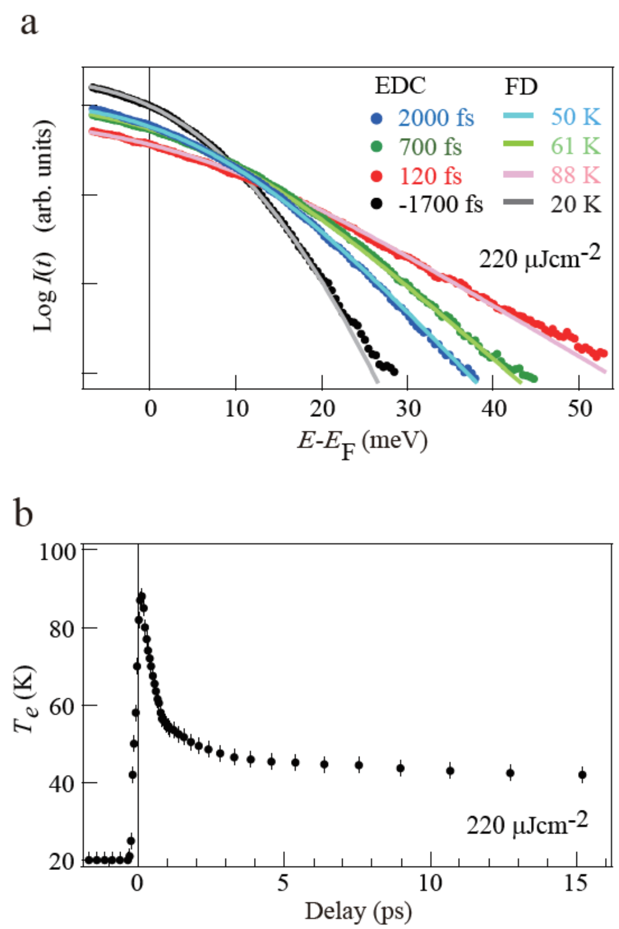

Supplementary Fig. 6. Estimation of the electronic temperature. a, Transient EDCs for *xz* and FD functions assuming a constant density of states. **b**, Time dependence of the electronic temperature for *xz* at 220 μJcm^{-2}.

Supplementary References:

[1] Suzuki, Y. *et al. Phys. Rev. B* **92**, 205117 (2015).

[2] Shimojima, T. *et al. Phys. Rev. B* **90**, 121111(R) (2014).

[3] Anisimov, S.I. *et al. J. Exp. Theor. Phys.* **66**, 375 (1974).

[4] Patz, A. *et al. Nature Communications* **5**, 3229 (2014).
